# What Is Needed to Encourage Women to Choose Careers in Interventional Cardiology

**DOI:** 10.1016/j.jscai.2024.102252

**Published:** 2024-09-20

**Authors:** Ariel Roguin, Holly Bauser-Heaton

**Affiliations:** a Cardiology Department, Hillel Yaffe Medical Center, Hadera, Israel; b Rappaport Faculty of Medicine, Technion – Israel Institute of Technology, Haifa, Israel; c Department of Pediatrics, Children's Healthcare of Atlanta Cardiology, Atlanta, Georgia

**Keywords:** catheterization laboratory, policy, radiation, safety, women

In recent years, the number of women studying medicine has increased, and they now constitute >50% of all medical students. Women can be seen today in all fields of medicine, and they make up 42% of internal medicine trainees. When it comes to cardiology fellowships, however, the numbers decrease, and women comprise only a small percentage of physicians in the field of fluoroscopically-guided interventions, such as electrophysiology, interventional radiology, and interventional cardiology.^1^ Among women younger than 40 years of age, the most commonly identified barrier to not choosing careers in these areas was radiation exposure and potential hazards to the fetus.

Interestingly, although there are no differences in pregnancy and radiation exposure worldwide, significant geographical practice variations exist ranging from forced absence from the catheterization laboratory to almost full working privileges during pregnancy.^2^ Strict regulations and rules for radiation exposure during pregnancy are not based on a large body of data, and evidence for these dose thresholds is based mainly on studies on large-scale radiation disasters,^1^ very small animal studies, and outcomes in women exposed to radiation for medical treatment.^2^ Scarce data result in the consideration of the principle of “primum non nocere”—above all, do no harm! This results in a default policy of limiting pregnant individuals from participation in the cardiac catheterization laboratory, creating a large gap in the number of women choosing to work in catheterization laboratories around the world, particularly if they sense regulations are based on knowledge to prevent potential harm. Yet the data for these regulations are weak.

Cheney et al^2^ recently published an extensive review summarizing the available data on occupational radiation exposure during pregnancy, with an emphasis on radiation quantification, the impact of exposure at various stages of fetal development, societal recommendations for safe levels of exposure during gestation, threshold levels necessary to induce fetal harm, and safe practices for the pregnant interventionist. They conclude that pregnancy in the cardiac catheterization laboratory is both safe and feasible. Similar conclusions were reached by other recent reviews on this field.^1^^,^^3^, ^4^, ^5^

The main issue is how to educate our professional society and decrease the distress and fear from the hazards of radiation, the amount we are exposed to, and the unfounded concern as to whether we are causing harm to our offspring. The education around this issue is crucial first for female cardiologists and staff, both in terms of making an informed decision about staying in the catheterization laboratory during pregnancy and knowing how to monitor and reduce their radiation exposure. Education should also be focused at the level of hospital management and catheterization laboratory directors so that decision-making can be data-driven. Finally, the education of policy/lawmakers is another important step to overcoming barriers to pregnancy in the catheterization laboratory.

Understanding available data and using appropriate protection may increase the number of women in the fields of interventional cardiology, electrophysiology, and interventional radiology. Understanding the radiation protection principles of time, distance, collimation, and shielding is critical to minimizing exposure. All measures should be used to decrease radiation exposure measures. The pregnant woman may require some additional specific measures (Table 1).^1^

**Table 1:** Specific strategies for radiation protection during pregnancy in the catheterization laboratory

Category	Strategy
1. Time	• Reduce time of procedures by avoiding unnecessary cines. • Minimize fluoroscopy time. Reduce unnecessary catheter manipulations under fluoroscopy. • Plan and study fluoroscopy-guided interventions in advance for cases with previous interventions or previous cines.
2. Source	• In EP, apply all technologies that allow to perform procedures without radiation. • Use electro-anatomical mapping systems in EP whenever possible. • Obtain appropriate training. • Use the lowest possible frame rate. • Use low magnification. • Avoid cine acquisition by using the “fluoro-save” option. • Use of collimation. • Use short intermittent radiation instead of continuous.
3. Distance	• Place the image detector as close as possible to the patient, to reduce scatter radiation. • Use available patient dose reduction technologies. • Wear your dosimeters and know your own dose. • Avoid hands in the primary beam. • Avoid being close to the x-ray tube. • Minimize the use of extremely angulated projections. • Minimize the use of projections with higher effective doses to the operator such as left anterior oblique-cranial.
4. Shielding	• Always keep the x-ray tube under the table. • Protection devices: lead glass goggles, thyroid protection, 2-piece lead apron (jacket and skirt) to distribute weight and 0.5 mm equivalent width (overlapping 2 layers of lead) at the front and 0.25 mm on the back (>90% protection). • Ancillary shields: Ceiling mounted screen, table curtain, lead skirt over the patient´s waist and mobile floor shield, especially during cine acquisition.
5. Specific measures for fertility in women	• Radiation cabin: Zero-gravity (Biotronik) and Cathpax Air (LemerPax) • Double thickness of abdominal shielding. • Improve work practices in the catheterization laboratory before pregnancy. • Continue with the same practices as before the pregnancy but with greater care. • Position yourself in a low-scatter area.

Adapted from Saada et al.^1^

EP, electrophysiology.

There are reports describing the personal experiences of pregnant women who have continued their careers and practice of interventional cardiology procedures and report safe radiation threshold results.

In a retrospective study of 758 neurointerventional cases (47 cases/month before pregnancy and 46 cases/month during pregnancy, *P* = .862) of a fellow physician wearing an additional dosimeter under the apron in the middle of the lower abdomen and 2 lead apron skirts showed 0 fetal radiation exposure without significant changes in the fellow´s case volume and fluoroscopy time.^6^ A case series of 5 operators from Spain, performing interventional procedures throughout pregnancy, found that the abdominal dose was well below the reference limit and in some cases no higher than the background radiation level.^7^

In the current issue of *JSCAI*, Fetterly et al^8^ from the Mayo Clinic present data on this important topic of radiation hazards and pregnancy. They conclude that radiation exposure to an assumed pregnant cardiologist can be maintained at low, safe levels and that these data may further embolden women to pursue careers in invasive cardiology. The authors tested among 42 physicians radiation exposure to the abdomen area which transmits through the protective garment material of invasive cardiologists. This can contribute to hypothetical fetus dose during pregnancy. The exposure was monitored during a 6-month period among both female and male interventional cardiologists and electrophysiologists during their clinical routine. Exposure to the abdomen of invasive cardiologists increased proportional to patient exposure and decreased predictably with increasing radioprotective material thickness. The median abdomen exposure when covered by 0.5 mm Pb equivalent radioprotective material over a 40-week period was only 0.22 mGy. These values are far below the current thresholds allowed. Interestingly, physician sex, height, and clinical role did not influence occupational exposure.

With this in mind, we must also improve the environment in which interventionalists work.^9^ Working with 2 layers of lead during pregnancy increases the weight on the spine and lower limbs which, in itself, only exacerbates the strain noted during gestation and additionally influences the health effects of the pregnant woman, namely venous insufficiency, thromboembolic risk, etc. An ill-fitting lead apron that does not cover the full axillary area may lead to increased ionizing radiation to the breast. During pregnancy and breastfeeding, the mammary glands are particularly sensitive to the effects of ionizing radiation. Properly fitting preventive equipment is key and necessary for women in our field.

Although the evidence of the effects of radiation on infertility, miscarriage, fetal risk, and birth defects is limited, the available data point toward a very low risk of adverse events.^1^, ^2^, ^3^, ^4^, ^5^^,^^10^ Lack of accurate knowledge regarding the risk of occupational radiation exposure before conception and during pregnancy can cause fear leading to anxiety and altered career and family choices. Data reveal female cardiologists made more changes in their training and careers to reduce or avoid radiation exposure because of concerns related to risk to a developing fetus.

Fear of radiation exposure during childbearing years disproportionately worries women from entering the field and many women follow the practice of “better be safe than sorry;” however, we should all be reminded about several important facts regarding pregnancy. Even in the general population, without any exposure to radiation, the success rate of becoming pregnant within a year is 80% to 85% and declines with age. Spontaneous abortion, mainly in the first trimester, occurs in approximately 15% of known pregnancies. Approximately 2% to 4% of babies are born with congenital malformations or abnormalities that are usually so mild that they are, at times, not even diagnosed. Although multifactorial, the rate of learning disability and disorders, especially attention and concentration problems, are among the most common behavior control difficulties exhibited by children and adolescents, and their rates have increased significantly in recent decades. Therefore, even without exposure to radiation, pregnancy is not always straightforward, and the additional exposure to radiation makes risk attribution complex, and not all complications are related to radiation exposure. That said, all available strategies should be used for radiation protection to reduce exposure and the potential risk for childbearing and pregnancy. Data suggest this exposure is very low and should not be the reason for women to be deterred from working and choosing careers in interventional cardiology. With better education, consistent messaging, improved understanding of radiation physics and hazards, and a better radiation protection environment,^9^ more women may be expected in the future to choose careers in interventional cardiology.

## References

[1] Saada M., Sanchez-Jimenez E., Roguin A. (2023). Risk of ionizing radiation in pregnancy: just a myth or a real concern?. Europace.

[2] Cheney A.E., Vincent L.L., McCabe J.M., Kearney K.E. (2021). Pregnancy in the cardiac catheterization laboratory: a safe and feasible endeavor. Circ Cardiovasc Interv.

[3] Manzo-Silberman S., Velázquez M., Burgess S. (2023). Radiation protection for healthcare professionals working in catheterisation laboratories during pregnancy: a statement of the European Association of Percutaneous Cardiovascular Interventions (EAPCI) in collaboration with the European Heart Rhythm Association (EHRA), the European Association of Cardiovascular Imaging (EACVI), the ESC Regulatory Affairs Committee and Women as One. EuroIntervention.

[4] Albakri A.A., Alzahrani M.M., Alghamdi S.H. (2024). Medical imaging in pregnancy: safety, appropriate utilization, and alternative modalities for imaging pregnant patients. Cureus.

[5] Englander M.J., Ghatan C. (2021). Radiation and the pregnant IR: myth versus fact. Cardiovasc Intervent Radiol.

[6] Chen S.H., Brunet M.-C. (2020). Fetal radiation exposure risk in the pregnant neurointerventionalist. J Neurointerv Surg.

[7] Velázquez M., Pombo M., Unzué L., Bastante T., Mejía E., Albarrán A. (2017). Radiation exposure to the pregnant interventional cardiologist. Does it really pose a risk to the fetus?. Rev Esp Cardiol (Engl Ed).

[8] Fetterly K.A., Schueler B.A., Mihailovic J.M. (2024). Radiation exposure and protection for (assumed) pregnant interventional cardiologists and electrophysiologists. J Soc Cardiovasc Angiogr Interv.

[9] Roguin A., Wu P., Cohoon T. (2023). Update on radiation safety in the cath lab – moving toward a “lead-free” environment. J Soc Cardiovasc Angiogr Interv.

[10] Dauer L.T., Miller D.L., Schueler B. (2015). Occupational radiation protection of pregnant or potentially pregnant workers in IR: a joint guideline of the Society of Interventional Radiology and the Cardiovascular and Interventional Radiological Society of Europe. J Vasc Interv Radiol.

